# The Assessment of Static Balance in Patients after Total Hip Replacement in the Period of 2-3 Years after Surgery

**DOI:** 10.1155/2018/3707254

**Published:** 2018-01-04

**Authors:** Teresa Pop, Daniel Szymczyk, Joanna Majewska, Agnieszka Bejer, Joanna Baran, Arkadiusz Bielecki, Wojciech Rusek

**Affiliations:** ^1^Institute of Physiotherapy, Faculty of Medicine, University of Rzeszów, Rzeszów, Poland; ^2^The Holy Family Specialist Hospital, Rudna Mała, Poland; ^3^Rehabilitation Center REHAMED-CENTER, Tajęcina, Poland

## Abstract

**Introduction:**

The aim of this study was to assess static balance of patients after Total Hip Replacement (THR) compared with the age-matched, asymptomatic control group, considering the subject's gender and the time since the surgery.

**Materials and Methods:**

The Total Hip Replacement (THR) group consisted of 55 subjects (mean age: 56.3 ± 8.7 years) and the control group consisted of 48 subjects (mean age: 58 ± 6.2 years). For the assessment of static balance, a stabilometric force platform was used. All subjects performed two 30-second trials in the double-leg stance position with eyes opened and closed. In the study group, the stabilometric assessment was performed once within the period of 24 to 36 months after the surgery.

**Results:**

Subjects from the study group had significantly increased mediolateral COP velocity in the test with eyes opened, as well as the values of most of the COP parameters (excluding COP path area) in the test with eyes closed, compared to the control group. Higher values of the selected COP parameters were observed in the male subjects from the study group.

**Conclusion:**

In contrast to a number of papers, our study revealed some deficits in static balance in patients after THR up to 2-3 years after surgery.

## 1. Introduction

Osteoarthritis (OA), mainly of the hip and knee joints, is the major joint disease among elderly people [1, 2]. The symptoms of hip OA, such as pain and stiffness of the joints, lead to impaired hip muscle strength and a restricted range of motion [3–5]. Since the decreased muscle strength and the impaired balance are important fall risk factors in older adults [6, 7], the evaluation of balance and postural control in the group of hip OA patients is very important in clinical practice.

Total Hip Replacement (THR) is a commonly performed surgical procedure in the case of advanced hip OA. Even though THR is very effective in achieving its primary objective of relieving pain, some functional limitations may persist after surgery [8–10]. After total hip arthroplasty (THA), the ability to perform activities of daily living (ADL) generally improves, but some activities may still be challenging and difficult [11–14].

The outcome of THA is traditionally measured with clinical scoring systems such as the Merle d'Aubigné-Postel score and the Harris hip score [15, 16]. These scoring systems are subjective and do not include objective measurements of a subject's functional status. Thus, in order to support an evidence-based approach, as well as to obtain a complex objective evaluation of function after hip surgery, it is necessary to use quantitative measurement techniques such as gait analysis or static and dynamic posturography [17–19].

The ability to maintain a stable, upright posture is an essential component of daily activities. Postural control is a complex function controlled by sensory input, central processing, and neuromuscular responses. Sensory components include the vestibular, visual, and different somatosensory information from the receptors in the joint capsules, ligaments, and surrounding muscles, as well as from the skin mechanoreceptors [20, 21]. Pathologies in the joint and the surrounding soft tissue may affect the quality of sensory information, resulting in decreased proprioception and disrupting the automatic postural responses to sensory inputs [21–23]. The effective motor response requires sufficient muscle strength and proper neuromuscular system function, which is essential to maintain balance and postural stability, and their impairment is an important factor increasing fall risk, especially in the group of older adults suffering from joint diseases [23–25].

The hip joint plays a pivotal role in stabilizing the body and maintaining postural balance. The “hip strategy” has been described as a compensatory mechanism to control the horizontal position of the body centre of mass at the hip level [26]. Older adults (over 60 years) predominantly use hip muscles to maintain static postural balance, because their muscular strength and distal proprioceptive inputs decrease with ageing [27]. Hip osteoarthritis (HOA) and further impairment of the hip muscles by the surgery can therefore strongly influence the role of the hip joint in maintaining balance. Joint stiffness and the increase of joint reactive forces can also lead to increased muscle energy expenditure and muscle fatigue [28].

According to the literature, postural control disorder persists from 3 to 8 weeks after surgery, due to muscle damage and residual pain [18, 29–32]. Holnapy and Kiss have evaluated balancing ability in response to sudden unidirectional perturbation changes during the first 6 months of the postoperative period in patients after THA, with respect to different methods of joint exposure during the operation. They showed that in case of direct-lateral and anterolateral exposure the dynamic balancing ability continuously improved in the first 6 months of the postoperative period, but the dynamic balancing ability of the affected limb differed from the control group. However, there was no significant difference in the balancing ability at 6 months after total hip arthroplasty in patients with posterior exposure during a total hip arthroplasty [33].

On the other hand, Rasch et al. have demonstrated a slow morphological recovery in OA, manifesting as a deficit in the cross-sectional area (CSA) and radiological density (RD) of hip muscles compared to a healthy limb up to 2 years after THA [34]. These morphological findings and the fact that patients who had undergone hip arthroplasty have shown a persistent deficit of hip muscles strength up to 2-3 years after THA [35, 36], as well as the small number of long-term results regarding postural control in patients after THR, have encouraged us to conduct this scientific research.

## 2. Paper's Purpose

The aim of this study was to assess the static balance of patients after a Total Hip Replacement (THR) compared to the age-matched healthy control group, considering the subject's gender and the time since the surgery. We hypothesised that impaired static balance can be present up to 24 to 36 months after THA, compared to the healthy control group.

## 3. Materials and Methods

This study was approved by the Bioethical Committee at the Medical Faculty of the University of Rzeszów. All subjects were informed about the procedures used in this study and signed a written informed consent.

### 3.1. Participants

Fifty-five patients (31 females and 24 males, mean age: 56.3 ± 8.7 years, mean BMI: 27.3 ± 4.1) with advanced, unilateral hip osteoarthritis who were treated by THR were included in the study group. Fifteen patients, out of seventy initially enrolled in this study, were excluded for not meeting the study group inclusion criteria. All patients in the study group were expected to maintain an upright standing position without any aid or physical assistance for the purpose of static balance assessment on the stabilometric force platform. The data obtained from THR patients were compared with the control group which consisted of 48 age-matched, healthy volunteers (31 females and 17 males, mean age: 58 ± 6.2 years, mean BMI: 26.5 ± 3.3). Twenty-two subjects from the control group, out of seventy initially invited to participate in this study, were excluded for not meeting the inclusion criteria. None of the subjects in the control group had any hip pain or functional impairment of the hip joint. There were no statistically significant differences in the structure of the study and control groups taking into account the subjects' age and gender.

All patients had undergone unilateral THR surgery with a lateral approach and uncemented prosthesis. All the surgical procedures were a minimally invasive (joint capsule saving) approach performed by the same team of orthopedic surgeons in the same clinic (the Holy Family Specialist Hospital, Rudna Mała, Poland) using the same implant type. None of the subjects in the study group had any early postsurgical complications.

The following postoperative protocol was performed: day 1: active and passive mobilisation of the limb; day 2: drainage removal; and day 3: assisted walking. The postsurgery rehabilitation program consisted of 4-week standard protocol including progressive strengthening exercise of the hip muscles, progressive range of motion (ROM) exercise, stretching of the hamstrings and hip adductor muscles, progressive weight-bearing walking with crutches, and the educational program focused on the potential postsurgical complications and limitations (including nursing and ADL instructions).

Static balance assessment, using the stabilometric force platform, was performed once between 24 and 36 months after the THR surgery (mean time since surgery: −29,2 months; min: −24 months; max: −36 months).

The exclusion criteria for both the study and control groups were as follows: lack of the subject's written consent to participate in the study, subjects above 65 years of age, severe obesity (BMI > 35), any other surgical procedure in the previous 12 months, rheumatological disease, history of spinal surgery, and lower limb fractures. We have also excluded patients who had any complaints regarding other lower limb joints or impairments of the lumbosacral area and subjects with visual or vestibular system impairments or any disease that could affect their static balance ability, such as cancer, endocrine disorders, cerebrovascular disease, Parkinson's syndrome, epilepsy, polyneuropathia, neuromuscular disorder, uncontrolled cardiovascular disease, or atherosclerosis of the lower limbs.

### 3.2. Static Balance Assessment

Static balance was assessed using a stabilometric force platform (Alfa, AC International East, Poland). The acquisition frequency was 62 Hz, which was recommended as an adequate sampling frequency for static balance evaluation [37]. Calibration of the stabilometric platform was conducted prior to the data acquisition based on the manufacturer's instructions. All subjects were asked to stand barefoot on the force platform facing the anteroposterior (AP) direction, in a comfortable, self-chosen, double-leg stance position, with their arms alongside the body. They were instructed to stand as still as possible during the tests and to breathe normally. Before the first test, the outline of each participant's feet was recorded to ensure a constant foot position between trials [38]. Data were collected in both visual conditions (eyes opened and eyes closed) in random order. During the test with opened eyes, subjects were asked to look straight ahead at a visual reference point (a red dot, 3 cm in diameter, placed 2 meters away on the wall, at eye height). Each test lasted for 30 seconds, followed by 30 seconds of rest interval. All subjects were asked to assume a testing position and the data acquisition started after they declared their readiness for testing. Additional trials were performed in the case of loss of balance, with 3 attempts allowed per testing condition. The following center of pressure (COP) parameters acquired during the tests with eyes opened (EO) and eyes closed (EC) on the stabilometric platform were used for the purpose of the subject's static balance evaluation: COP deviation mean velocity in anterior-posterior (AP) and medial-lateral (ML) directions (cm/s), COP path length (cm), and COP path area (cm^2^), as well as the Romberg test results for COP path length and COP path area (EC/EO ratio for the COP path length and COP path area, resp.).

### 3.3. Data Analysis

To evaluate the significance of the differences in the selected stabilometric parameters between the study and control groups, a nonparametric Mann–Whitney test was used. The significance of the differences between the results of the stabilometric tests with eyes opened and closed was calculated using a nonparametric Wilcoxon test. The impact of the time since the surgery on the selected stabilometric parameters was assessed using a Spearman's rank correlation coefficient, while the gender differences of the stabilometric parameters were calculated using the Mann–Whitney test. The level of significance was assumed at *α* < 0.05. All calculations and statistical analysis were performed using STATISTICA ver. 10.0 (StatSoft, Poland).

## 4. Results

There were statistically significant differences in the COP deviation mean velocity in ML direction between the study and the control groups during the test with eyes opened (EO). Higher values for both of these parameters were present in the study group. More significant differences were observed during the test with eyes closed (EC). All stabilometric parameter values, excluding the COP path area, were significantly higher in the study group (Table 1).

We also observed some expected, statistically significant differences concerning the same stabilometric parameters between the tests with eyes closed and eyes opened, both in the study group and in the control group. Disabling of the visual input in the eyes closed testing condition may lead to expected increase of the subjects' postural sway.  Table 2 presents basic descriptive statistics concerning the difference in the results between eyes closed and eyes opened trials, in the control and study groups, separately. For all the analyzed stabilometric parameters, the value of the differences between the results obtained in the eyes closed versus eyes opened trials was higher in the study group (Table 2).

However, there were no statistically significant differences in the results of the Romberg test (EC/EO ratio for the COP path length and COP path area, resp.) between the study and the control groups (Table 3).

Considering the subjects' gender, no statistically significant differences were observed for the selected COP parameters value between women and men in the control group. However, there were statistically significant differences between women and men in the study group during the test with eyes opened. Significantly higher values of stabilometric parameters were observed in male subjects in the study group (Table 4).

Table 5 presents the differences in the stabilometric parameters between the study group and control group, separately for men and women. There were statistically significant differences in most of the analyzed parameters (excluding COP path area and EC/EO ratio) between male subjects from the study and control groups, in both testing conditions (EO and EC). The results for male subjects from the study group were higher than the analogical results of the men from the control group, although, we have not observed such a statistically significant difference between female subjects from the study and control groups.

Considering the period of time since the surgery, we can observe that this variable is correlated with some improvement of the stabilometric tests result (decrease of the COP parameters value), especially during the test with eyes opened (EO). We have found some significant, negative correlations considering most of the stabilometric parameters during eyes opened trials (EO) in the study group; however, these correlations were not very strong (Table 6).

## 5. Discussion

Functional and clinical outcomes in patients after THR are reported to be quite positive, considering the improvement in hip muscles isometric strength, the improvement of the gait velocity, and the improved quality of life in the early postsurgical period [10, 39, 40]. Despite these positive outcomes, previous studies have reported some deficits in postural control up to 6–12 months after surgery [30, 31]. To our knowledge, there are no quantitative studies of static balance in patients after THR concerning long-term results in this population. This is also important due to the fact that patients after hip arthroplasty have shown a persistent deficit of hip muscles strength up to 2-3 years after THR [35, 36]. The purpose of this study was therefore to evaluate the static balance in patients after THR to determine whether 24–36 months after surgery the stabilometric parameters are similar to those of the asymptomatic, age-matched control group. In our study, all subjects performed a stabilometric test on a force platform in both visual conditions: with eyes opened and closed. Objective measures of static balance and postural control using computerized force platforms are considered to be current “gold standards” for clinical and scientific practice [41].

In the process of maintaining static balance and postural control, visual input plays a significant role. Therefore, we can expect that the lack of visual information leads to an increased postural sway [42]. In our study, we also observed a significant difference in the COP deviation mean velocity in ML direction between the study and control groups during the test with eyes opened (EO). More significant differences between the study and control groups, concerning most of the stabilometric parameters (excluding the COP path area), were revealed during the test with eyes closed (EC). This may indicate that the lack of visual input during the eyes closed (EC) test emphasizes the proprioceptive deficits in the patients after THR and increase the patients' postural sway compared to the asymptomatic control group.

Considering the period of time between the surgery and the stabilometric assessment, we observed some improvement of the result in the study group (decrease of the values of the stabilometric parameters depending on the time since surgery), although there were still some significant differences compared to the control group. These findings, considering the fact that all patients in our study were evaluated between 24 to 36 months after surgery, are in contrast to the results of similar studies reported by other authors. Wykman and Goldie evaluated twenty-one patients before and one year after Total Hip Replacement surgery. They found that the patient's static balance improved and the postural sway values were similar to those observed in healthy people [43]. Similarly, Calo et al. performed a dynamic posturography assessment in a group of patients with THR four months after surgery and reported a lack of difference compared with age- and gender-matched control subjects [19]. On the other hand, Nallegowda et al. performed dynamic posturography tests in patients with THR nine months after the surgery, and their results indicate that there are no proprioceptive deficits in this group but some delayed motor responses are still present [44]. Nantel et al. assessed postural balance in patients after THR and after surface replacement arthroplasty (in both cases six months after surgery) as well as in the control subjects. The results of their study showed a greater center of pressure and center of mass displacement amplitude in the medial-lateral direction during the double-leg stance for the total hip arthroplasty group compared to the surface replacement and control subjects. This indicates that the better anatomical preservation and the absence of femoral stem in the surface replacement patients group may result in a better static balance and postural control in comparison to patients with THR [45]. For this reason, while comparing the results of such studies to those obtained by other researchers, it is suggested that homogenous groups of patients should be compared. However, due to the large number of variables (i.e., many types of prostheses, various surgical techniques, and anatomical conditions), this approach seems to be difficult to use in scientific and clinical practice.

Considering the patients' gender, in our study group, we observed significantly higher values of the selected COP parameters in male subjects, compared to female subjects. Besides, separate statistical analysis of the results for men and women revealed some statistically significant difference between male subjects from the study and the control group, while we have not observed such differences between women from the study and control groups. This may indicate that analysis of such results should be done separately for men and women. Moreover, considering lack of the stabilometric data normal distribution and relatively high variability, as well as the very complex nature of the human balance and postural control abilities, we can assume that analyzing and precise interpretation of such results are difficult and some important factors, such as patients' age, gender, and overall health status, should be taken into account.

There are some limitations of our study. One of the important limitations is the fact that only static balance was assessed in our study and we did not assess the patient's dynamic balance, which seems to be more functional. The other limitation is lack of the patients' static balance assessment at the earlier stages after THR, which can be useful to observe the changes of the stabilometric parameters in time.

## 6. Conclusions 

Static balance parameters in the group of THR patients can still be impaired up to 2-3 years after the surgery, compared to the age-matched, asymptomatic control group.

Static balance assessment during the test with eyes closed seems to be more sensitive for detecting proprioceptive deficits in this group of patients, compared to the control group.

The assessment of balance and postural control should be an integral part of a complex evaluation and monitoring of a patient's functional status at different stages after THR surgery, considering the increased fall risk in this group.

## Figures and Tables

**Table 1:** Stabilometric parameters (eyes opened (EO) and eyes closed (EC)): study group versus control group.

	Eyes opened (EO)										*p*
	Control group					Study group					
	M	Me	SD	Min	Max	M	Me	SD	Min	Max	
COP deviation mean velocity (AP) (cm/s)	0,44	0,41	0,19	0,18	1,06	0,51	0,45	0,27	0,15	1,87	0,147
COP deviation mean velocity (ML) (cm/s)	0,49	0,43	0,23	0,18	1,42	0,59	0,53	0,29	0,17	1,64	0,037
COP path length (cm)	20,76	18,46	9,53	7,76	55,82	24,64	22,09	12,69	7,44	79,69	0,071
COP path area (cm^2^)	1,77	1,09	2,48	0,36	16,94	2,02	1,23	1,88	0,27	9,58	0,264

	Eyes closed (EC)										*p*
	Control group					Study group					
	M	Me	SD	Min	Max	M	Me	SD	Min	Max	

COP deviation mean velocity (AP) (cm/s)	0,76	0,68	0,35	0,31	1,95	0,90	0,88	0,37	0,31	2,50	0,026
COP deviation mean velocity (ML) (cm/s)	1,00	0,80	0,64	0,33	4,15	1,34	1,29	0,66	0,35	3,05	0,001
COP path length (cm)	40,32	33,71	23,42	14,57	145,2	52,36	51,28	24,82	14,44	122,7	0,005
COP path area (cm^2^)	3,99	2,86	5,19	0,50	29,66	4,31	3,36	3,56	0,39	18,19	0,190

AP: anterior-posterior; COP: center of pressure; EC: eyes closed; EO: eyes opened; M: mean; max: maximum value; Me: median; min: minimum value; ML: medial-lateral; *p*: Mann–Whitney test probability value; SD: standard deviation.

**Table 2:** Differences in the stabilometric parameters between the tests with eyes closed and eyes opened: control and study group.

Stabilometric parameters (EC versus EO)	Control group						Study group					
	M	Me	SD	Min	Max	*p*	M	Me	SD	Min	Max	*p*
COP deviation mean velocity (AP) (cm/s)	0,32	0,31	0,25	−0,17	1,31	≤0,001	0,39	0,36	0,23	−0,16	1,10	≤0,001
COP deviation mean velocity (ML) (cm/s)	0,51	0,41	0,54	−0,07	3,45	≤0,001	0,75	0,61	0,51	−0,20	2,12	≤0,001
COP path length (cm)	19,56	17,24	18,7	−6,82	115,58	≤0,001	27,72	24,36	18,0	−9,56	76,49	≤0,001
COP path area (cm^2^)	2,22	1,47	4,17	−2,46	27,32	≤0,001	2,29	1,48	3,27	−7,29	12,91	≤0,001

AP: anterior-posterior; COP: center of pressure; EC: eyes closed; EO: eyes opened; M: mean; max: maximum value; Me: median; min: minimum value; ML: medial-lateral; *p*: Wilcoxon test probability value; SD: standard deviation.

**Table 3:** The Romberg test results (EC/EO ratio): study group versus control group.

The Romberg test (EC/EO ratio)	Control group					Study group					*p*
	M	Me	SD	Min	Max	M	Me	SD	Min	Max	
COP path length (cm)	1,99	1,94	0,73	0,80	4,90	2,26	2,09	0,84	0,60	4,60	0,117
COP path area (cm^2^)	2,66	2,31	2,02	0,53	12,70	2,88	2,25	2,62	0,24	16,89	0,940

COP: center of pressure; EC: eyes closed; EO: eyes opened; M: mean; max: maximum value; Me: median; min: minimum value; *p*: Mann–Whitney test probability value; SD: standard deviation.

**Table 4:** Gender differences in the stabilometric parameters in the study group and the control group.

Stabilometric parameters	Study group						*p*
	Men			Women			
	M	Me	SD	M	Me	SD	
EC/EO ratio							
COP path length (cm)	2,03	1,96	0,70	2,43	2,26	0,91	0,047
COP path area (cm^2^)	2,61	2,29	1,88	3,09	1,97	3,09	0,730

Eyes opened (EO)							
COP deviation mean velocity (AP) (cm/s)	0,62	0,57	0,33	0,42	0,40	0,16	≤0,001
COP deviation mean velocity (ML) (cm/s)	0,73	0,65	0,33	0,48	0,46	0,21	≤0,001
COP path length (cm)	30,43	26,83	14,9	20,16	19,03	8,50	0,001
COP path area (cm^2^)	2,38	1,92	1,66	1,75	1,11	2,01	0,015

Eyes closed (EC)							
COP deviation mean velocity (AP) (cm/s)	0,98	0,97	0,39	0,84	0,77	0,35	0,173
COP deviation mean velocity (ML) (cm/s)	1,44	1,38	0,59	1,26	1,05	0,71	0,129
COP path length (cm)	57,40	56,29	23,60	48,45	38,80	25,41	0,088
COP path area (cm^2^)	4,95	3,49	3,19	3,81	2,36	3,80	0,076

Stabilometric parameters	Control group						*p*
	Men			Women			
	M	Me	SD	M	Me	SD	

EC/EO ratio							
COP path length (cm)	1,89	1,88	0,58	2,04	1,96	0,81	0,685
COP path area (cm^2^)	2,65	1,99	1,66	2,67	2,32	2,22	0,898

Eyes opened (EO)							
COP deviation mean velocity (AP) (cm/s)	0,46	0,39	0,20	0,43	0,41	0,18	0,765
COP deviation mean velocity (ML) (cm/s)	0,51	0,42	0,27	0,47	0,44	0,20	0,669
COP path length (cm)	21,91	17,92	10,81	20,13	18,89	8,88	0,608
COP path area (cm^2^)	2,34	0,92	4,02	1,45	1,22	0,83	0,417

Eyes closed (EC)							
COP deviation mean velocity (AP) (cm/s)	0,76	0,64	0,31	0,77	0,69	0,37	1,000
COP deviation mean velocity (ML) (cm/s)	0,98	0,79	0,45	1,01	0,80	0,73	0,701
COP path length (cm)	39,40	33,61	17,41	40,83	33,81	26,40	0,733
COP path area (cm^2^)	4,16	3,26	5,25	3,89	2,55	5,24	0,864

AP: anterior-posterior; COP: center of pressure; EC: eyes closed; EO: eyes opened; EC/EO ratio: eyes closed/eyes opened ratio; M: mean; Me: median; ML: medial-lateral; *p*: Mann–Whitney test probability value; SD: standard deviation.

**Table 5:** Differences in the stabilometric parameters in male subjects (study group versus control group) and female subjects (study group versus control group).

Stabilometric parameters	Men (control group)			Men (study group)			*p*
	M	Me	SD	M	Me	SD	
EC/EO ratio							
COP path length (cm)	1,89	1,88	0,58	2,03	1,96	0,70	0,743
COP path area (cm^2^)	2,65	1,99	1,66	2,61	2,29	1,88	0,947

Eyes opened (EO)							
COP deviation mean velocity (AP) (cm/s)	0,46	0,39	0,20	0,62	0,57	0,33	0,019
COP deviation mean velocity (ML) (cm/s)	0,51	0,42	0,27	0,73	0,65	0,33	0,004
COP path length (cm)	21,91	17,92	10,81	30,43	26,83	14,90	0,015
COP path area (cm^2^)	2,34	0,92	4,02	2,38	1,92	1,66	0,058

Eyes closed (EC)							
COP deviation mean velocity (AP) (cm/s)	0,76	0,64	0,31	0,98	0,97	0,39	0,021
COP deviation mean velocity (ML) (cm/s)	0,98	0,79	0,45	1,44	1,38	0,59	0,004
COP path length (cm)	39,40	33,61	17,41	57,40	56,29	23,60	0,006
COP path area (cm^2^)	4,16	3,26	5,25	4,95	3,49	3,19	0,143

Stabilometric parameters	Women (control group)			Women (study group)			*p*
	M	Me	SD	M	Me	SD	

EC/EO ratio							
COP path length (cm)	2,04	1,96	0,81	2,43	2,26	0,91	0,088
COP path area (cm^2^)	2,67	2,32	2,22	3,09	1,97	3,09	0,823

Eyes opened (EO)							
COP deviation mean velocity (AP) (cm/s)	0,43	0,41	0,18	0,42	0,40	0,16	0,769
COP deviation mean velocity (ML) (cm/s)	0,47	0,44	0,20	0,48	0,46	0,21	0,877
COP path length (cm)	20,13	18,89	8,88	20,16	19,03	8,50	0,988
COP path area (cm^2^)	1,45	1,22	0,83	1,75	1,11	2,01	0,674

Eyes closed (EC)							
COP deviation mean velocity (AP) (cm/s)	0,77	0,69	0,37	0,84	0,77	0,35	0,306
COP deviation mean velocity (ML) (cm/s)	1,01	0,80	0,73	1,26	1,05	0,71	0,076
COP path length (cm)	40,83	33,81	26,40	48,45	38,80	25,41	0,168
COP path area (cm^2^)	3,89	2,55	5,24	3,81	2,36	3,80	0,790

AP: anterior-posterior; COP: center of pressure; EC: eyes closed; EO: eyes opened; EC/EO ratio: eyes closed/eyes opened ratio; M: mean; Me: median; ML: medial-lateral; *p*: Mann–Whitney test probability value; SD: standard deviation.

**Table 6:** Correlation between stabilometric parameters and the time since surgery (eyes opened (EO) and eyes closed (EC)).

Stabilometric parameters	Time since surgery [months]
EC/EO ratio	
COP path length (cm)	0,02 (*p* = 0,898)
COP path area (cm^2^)	0,11 (*p* = 0,439)

Eyes opened (EO)	
COP deviation mean velocity (AP) (cm/s)	−0,30 (*p* = 0,026)
COP deviation mean velocity (ML) (cm/s)	−0,29 (*p* = 0,031)
COP path length (cm)	−0,29 (*p* = 0,029)
COP path area (cm^2^)	−0,24 (*p* = 0,079)

Eyes closed (EC)	
COP deviation mean velocity (AP) (cm/s)	−0,27 (*p* = 0,048)
COP deviation mean velocity (ML) (cm/s)	−0,22 (*p* = 0,102)
COP path length (cm)	−0,26 (*p* = 0,059)
COP path area (cm^2^)	−0,16 (*p* = 0,230)

AP: anterior-posterior; COP: center of pressure; EC: eyes closed; EO: eyes opened; EC/EO ratio: eyes closed/eyes opened ratio; ML: medial-lateral; *p*: Spearman's rank correlation coefficient.
